# Abnormal Expression of Proteolytic Stress-Related Proteins and Protective Effect of Fibrinolytic Enzymes in Prion Diseases

**DOI:** 10.1155/tbed/9527934

**Published:** 2025-02-26

**Authors:** Yong-Chan Kim, Sae-Young Won, Byung-Hoon Jeong

**Affiliations:** ^1^Department of Biological Sciences, Andong National University, Andong 36729, Republic of Korea; ^2^Korea Zoonosis Research Institute, Jeonbuk National University, Iksan 54531, Jeonbuk, Republic of Korea; ^3^Department of Bioactive Material Sciences and Institute for Molecular Biology and Genetics, Jeonbuk National University, Jeonju 54896, Jeonbuk, Republic of Korea

**Keywords:** biomarker, CJD, drug, endogenous protease, lumbrokinase, nattokinase, prion, proteolytic stress, scrapie, therapeutic potential

## Abstract

Prion diseases are fatal, irreversible, and infectious neurodegenerative diseases caused by proteinase K-resistant prion protein (PrP^Sc^). Against PrP^Sc^, several endogenous proteases involved in cellular degradation mechanisms can be activated to remove PrP^Sc^. However, since PrP^Sc^ shows proteinase K resistance, we presumed that undegradable PrP^Sc^ induces positive feedback on the overactivation of the cellular degradation mechanisms and is correlated with proteolytic stress and exacerbation of the progression of prion diseases. We investigated the expression pattern of proteolytic stress-related proteins in the brains of ME7 scrapie-infected mice at 7 months postinfection and sporadic Creutzfeldt–Jakob disease (CJD) patients using western blotting and immunohistochemistry (IHC). In addition, we analyzed the 3D structure and binding complexes of prion protein (PrP) with nattokinase and lumbrokinase using in silico programs, including SWISS-MODEL and HawkDock. To fundamentally reduce proteolytic stress by the degradation of PrP^Sc^, we performed an in vitro evaluation of the PrP^Sc^ degradation abilities of fibrinolytic enzymes, including nattokinase and lumbrokinase. Furthermore, we assessed the protective effects of nattokinase and lumbrokinase in ME7 scrapie-infected mice. We observed an abnormal accumulation of proteolytic stress-related proteins, including CD10, cathepsin B, cathepsin D, and matrix metalloproteinase 9 (MMP9), in the brains of ME7 scrapie-infected mice and sporadic CJD patients. In addition, we identified that nattokinase and lumbrokinase can stably bind to PrP. Furthermore, we identified significant in vitro degradation of PrP^Sc^ derived from ME7 scrapie-infected mice and sporadic CJD patients by nattokinase and lumbrokinase. Last, we found in vivo protective effects of nattokinase and lumbrokinase against prion disease in ME7 scrapie-infected mice. To the best of our knowledge, this is the first report on the identification of proteolytic stress-related novel potential biomarkers and the therapeutic potential of nattokinase and lumbrokinase for prion diseases.

## 1. Introduction

Prion diseases are fatal, irreversible, and infectious protein misfolded disorders (PMDs) caused by proteinase K-resistant prion protein (PrP^Sc^), which is conformationally converted from benign and normal prion protein (PrP^C^) [1–4]. Although prion diseases are classified according to host species, recent studies have reported several cases of infection that cross interspecies barriers against prion disease transmission [5–9]. In addition, the threat of prion diseases is increasing, but a clear therapeutic agent or vaccine has not been developed thus far, and understanding of the mechanism of prion disease remains insufficient [10, 11].

In prion diseases, several cellular mechanisms, including endoplasmic reticulum (ER)-mediated degradation (ERAD), unfolded protein response (UPR), and autophagy, can be activated to remove PrP^Sc^ [3, 12–14]. In recent studies, among effectors of those cellular mechanisms, several endogenous proteases play a crucial role in the direct degradation of misfolded and aggregated proteins, such as amyloid-beta and alpha-synuclein in Alzheimer's and Parkinson's diseases, respectively [15–17]. However, overactivation of these cellular mechanisms exacerbates disease progression and acts as a double-edged sword via proteolytic stress [18–20]. Indeed, since PrP^Sc^ showed proteinase K resistance, we presumed that undegradable PrP^Sc^ induces positive feedback on the overactivation of the cellular mechanisms and is associated with proteolytic stress and exacerbation of disease progression [21]. Thus, we screened proteolytic stress-related biomarkers and reduced the positive feedback loop driving the overactivation of proteolytic enzymes using exogenous proteases, such as nattokinase and lumbrokinase, which can degrade amyloid aggregates formed by the PrP106-126 peptide [22, 23].

In the present study, to identify novel proteolytic stress-related biomarkers in prion diseases, we investigated the expression pattern of proteolytic stress-related proteins in the brains of ME7 scrapie-infected mice at 7 months postinfection and sporadic Creutzfeldt–Jakob disease (CJD) patients using western blotting and immunohistochemistry (IHC). In addition, we predicted the 3D structure and binding complexes of murine prion protein (PrP) with nattokinase and lumbrokinase using in silico programs, including SWISS-MODEL and HawkDock [24, 25]. Furthermore, we carried out an in vitro evaluation of the PrP^Sc^ degradation capacity of fibrinolytic enzymes, including nattokinase and lumbrokinase [26, 27]. Finally, we evaluated the in vivo protective effects of nattokinase and lumbrokinase against prion disease in ME7 scrapie-infected mice by bioassay.

## 2. Materials and Methods

### 2.1. Ethical Statements

C57BL/6J mice were purchased from Nara Biotech (Pyeongtaek, Korea). All experimental procedures were accredited by the Institute of Animal Care and Use Committee of Jeonbuk National University (JBNU 2020-080). All samples of sporadic CJD patients and matched controls were obtained from the University of Edinburgh. All procedures were accredited by the IRB of Jeonbuk National University and followed the 1964 Declaration of Helsinki and its later amendments or comparable ethical standards (approval number: 2020-10-014).

### 2.2. Western Blotting

Total protein was extracted from brain tissues using RIPA lysis buffer (Thermo Fisher Scientific, Waltham, USA) containing a proteinase inhibitor cocktail (Roche, Munich, Germany). The samples were boiled at 95°C for 10 min in 5× sodium dodecyl sulfate (SDS) sample loading buffer (Thermo Fisher Scientific, Waltham, USA) and separated according to molecular weight in a 10%–15% SDS gel. The separated proteins were electrophoretically transferred to a nitrocellulose membrane (Amersham, Little Chalfont, UK) at 100 V for 1.5 h. The membranes were blocked with TBST (Thermo Fisher Scientific, Waltham, USA) containing 5% skim milk (Santa Cruz Biotechnology, Dallas, USA) for 1.5 h. The blocked membranes were incubated for 9 h with primary antibodies. Detailed information on the primary antibodies is described in Supporting Information 2: Table S1. The membranes were washed in TBST and incubated with horseradish peroxidase-conjugated secondary antibodies (Sigma–Aldrich, St. Louis, USA) for 1 h. After washing in TBST, the targeted proteins were visualized using a Pierce ECL kit (Thermo Fisher Scientific, Waltham, USA).

### 2.3. IHC

For immunohistochemical analysis, brain tissues were perfused with 1× phosphate-buffered saline (PBS) and fixed in 4% paraformaldehyde. The fixed brain tissues were serially transferred to a 10%–30% sucrose solution. Then, the brain was frozen in an optimal cutting compound (OCT). The embedded tissues were cut into 40 μm sections with a cryomicrotome (Thermo Fisher Scientific, Waltham, USA). The brain sections were heated in antigen retrieval reagent, pH 6.0 (Enzo Biochem, Inc., New York, USA) for 30 min. The brain sections were blocked with antibody blocker/diluent (Enzo Biochem, Inc., New York, USA) for 1 h. The blocked brain sections were incubated for 1 h with primary antibodies. Detailed information on the primary antibodies is presented in Supporting Information 2: Table S1. The brain sections were washed in PBST (Sigma–Aldrich, St. Louis, USA) and incubated with horseradish peroxidase-conjugated secondary antibodies (Enzo Biochem, Inc., New York, USA) for 1 h. After washing in PBST, the targeted proteins were visualized using 1× HighDef IHC chromogen substrate and working solutions (Enzo Biochem, Inc., New York, USA).

### 2.4. PrP^Sc^ Detection

To detect PrP^Sc^, 50 µg/mL proteinase K (Thermo Fisher Scientific, Waltham, USA; catalog number: BP1700-100) was added to quantified brain samples for 1 h at 37°C. The proteinase K-treated samples were boiled at 95°C for 10 min in 5× SDS sample loading buffer (Thermo Fisher Scientific, Waltham, USA). The proteinase K-treated samples were used to detect the PrP^Sc^ bands using western blotting.

### 2.5. In vitro PrP^Sc^ Degradation Assay

Proteinase K (50 µg/mL) was added to 100 μL of 2.5% (w/v) brain homogenate derived from terminally ill ME7 scrapie-infected mice and sporadic CJD patients. Then, proteinase K-treated samples were boiled at 95°C for 5 min and treated with nattokinase (Abmole, Houston, USA) and lumbrokinase (Creative Enzymes, New York, USA) for 5 h to evaluate the PrP^Sc^ degradation ability of these enzymes. A separate proteinase K-treated sample was treated with PBS for 5 h for the negative control. Each sample was used to detect the PrP^Sc^ bands by means of western blotting.

### 2.6. In Vivo Evaluation of the Protective Effect of Nattokinase and Lumbrokinase by Survival Analysis

The ME7 scrapie strain was obtained from The Roslin Institute, The University of Edinburgh. C57BL/6 mice (6 weeks old) were infected by intraperitoneal injection with 100 μL of 1% (w/v) brain homogenate derived from terminally ill ME7 scrapie-infected mice. C57BL/6 mice (6 weeks old) were injected via the intraperitoneal route with 100 μL of PBS for the negative control. One week postinjection, 100 μL nattokinase (5 mg/kg, *n* = 8) and lumbrokinase (20 mg/kg, *n* = 8) dissolved in 1× PBS were injected weekly to estimate the protective effect of these enzymes against prion disease. One week postinjection, 100 μL of 1× PBS (*n* = 7) was injected weekly as a negative control. At 5 months postinjection, the mice were sacrificed (three mice from each group), and PrP^Sc^ accumulation and astrocytosis were analyzed using western blotting. To carry out survival analysis, the remaining mice (nattokinase, *n* = 5; lumbrokinase, *n* = 5; PBS, *n* = 4) were observed daily postinjection until neurologic symptoms developed and were then sacrificed. Brains were obtained to detect PrP^Sc^ by western blotting. Kaplan–Meier survival analysis was performed with the survival (ver. 3.7-0) and survminer (ver. 0.4.9) packages of the R program (https://www.r-project.org/). Statistical significance was calculated using the log-rank test.

### 2.7. Statistical Analysis

Statistical analyses were carried out using SAS version 9.4 (SAS Institute Inc., USA). Data were recorded as the mean values ± standard deviations (SDs). More than three independent experiments were carried out. Statistical significance using the *p* value was calculated with a one-tailed Student's *t* test for single comparisons. The symbols *⁣*^*∗*^, *⁣*^*∗∗*^, and *⁣*^*∗∗∗*^ denote *p*  < 0.05, *p*  < 0.01, and *p*  < 0.001, respectively.

### 2.8. 3D Structure of Nattokinase and Lumbrokinase

The 3D structure of nattokinase was obtained from a protein data bank (PDB ID: 3VYV). The amino acid sequences of lumbrokinase were obtained from GenBank (Protein ID: AAL28118.1), and the 3D structure of lumbrokinase was predicted using the SWISS-MODEL program.

### 2.9. HawkDock

Protein–protein interactions and complex structures were analyzed using HawkDock [24, 25] based on the docking algorithm “ATTRACT.” The structure assessment of protein–protein interactions and binding free energy were estimated with MM/GBSA. The 3D structure of murine PrP (PDB ID: 1AG2) was deposited as the ligand. 3D structures of nattokinase and lumbrokinase were deposited as the receptors.

## 3. Results

### 3.1. Identification of Novel Proteolytic Stress-Related Potential Biomarkers in ME7 Scrapie-Infected Mice

**Figure 1:**
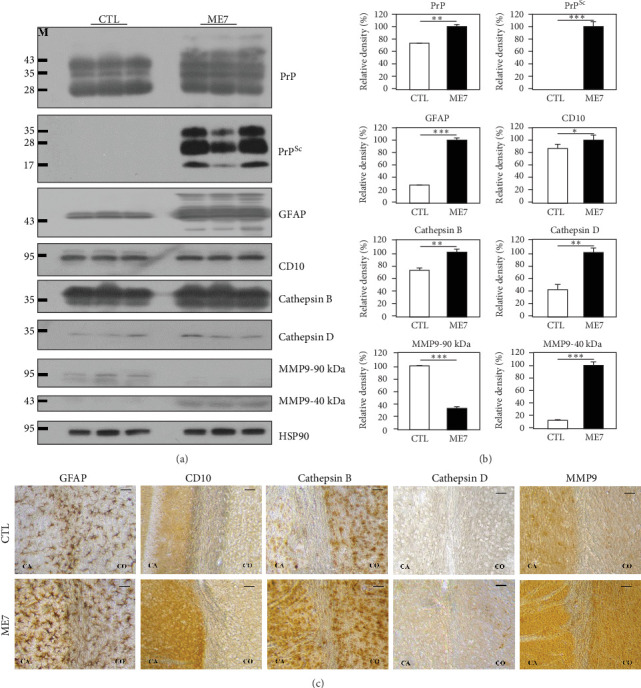
Altered expression of proteolytic stress-related proteins in ME7 scrapie-infected mice. (A) The expression patterns of proteolytic stress-related proteins were compared in whole brains between wild-type and ME7 scrapie-infected mice at 7 months postinjection using western blotting. (B) Quantification of the expression levels of proteolytic stress-related proteins was normalized to the HSP90 level. CTL: Phosphate-buffered saline (PBS)-inoculated mice, *n* = 3; ME7: ME7 scrapie-inoculated mice, *n* = 3. *⁣*^*∗*^*p*  < 0.05; *⁣*^*∗∗*^*p*  < 0.01; *⁣*^*∗∗∗*^*p*  < 0.001. (C) Histological analysis of proteolytic stress-related proteins in ME7 scrapie-infected mice. The expression patterns of proteolytic stress-related proteins were analyzed in the caudate putamen and cerebral cortex of wild-type and ME7 scrapie-infected mice at 7 months postinjection by immunohistochemistry (IHC). The upper panels indicate the IHC results in PBS-inoculated control mice. The lower panels indicate the IHC results in ME7 scrapie-inoculated mice. Images were taken using a microscope at 100x magnification. CA, caudate putamen; CO, cerebral cortex; N.S., not significant. Scale bar: 50 µm.

To identify novel proteolytic stress-related biomarkers in ME7 scrapie-infected mice, we compared the expression pattern of proteolytic stress-related proteins between ME7 scrapie-infected mice and matched controls at 7 months postinjection using western blotting (Figure 1A, B). In brief, total PrP and PrP^Sc^ were elevated and accumulated in ME7 scrapie-infected mice compared to control mice, respectively. In addition, alongside PrP^Sc^, a hallmark of prion diseases, astrocytosis marker glial fibrillary acidic protein (GFAP), was also significantly upregulated in ME7 scrapie-infected mice. Notably, proteolytic stress-related proteins, including CD10, cathepsin B, cathepsin D, and matrix metalloproteinase 9 (MMP9), showed altered expression patterns in ME7 scrapie-infected mice compared to matched controls. In detail, the expression levels of CD10, cathepsin B, cathepsin D, and MMP9-40 kDa were upregulated in ME7 scrapie-infected mice. However, MMP9-90 kDa was downregulated in ME7 scrapie-infected mice.

We also investigated the histological expression pattern of proteolytic stress-related proteins in the cerebral cortex and caudate putamen of ME7 scrapie-infected mice and matched controls at 7 months postinoculation by IHC (Figure 1C). Similar to the western blotting results, the astrocyte marker GFAP was upregulated in ME7 scrapie-infected mice. Notably, proteolytic stress-related proteins, including CD10, cathepsin B, cathepsin D, and MMP9, were also upregulated in the cerebral cortex and caudate putamen of ME7 scrapie-infected mice compared to matched controls.

### 3.2. Identification of Novel Proteolytic Stress-Related Biomarkers in Sporadic CJD Patients

**Figure 2:**
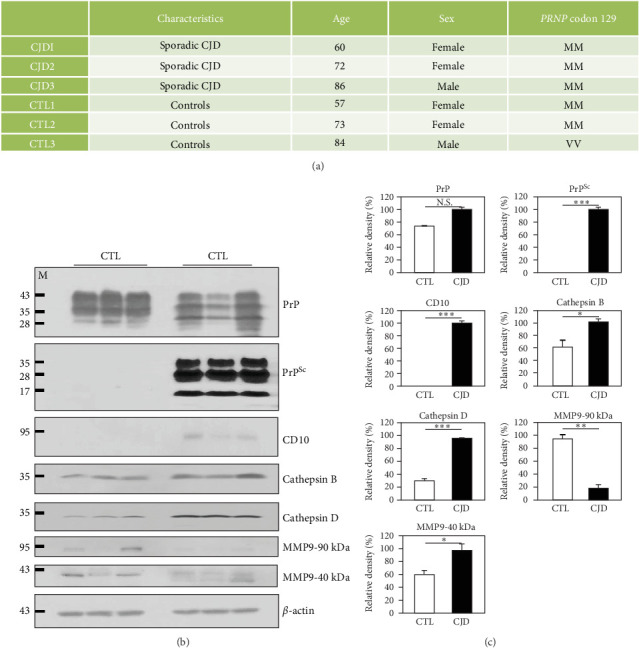
Altered expression of endogenous proteolytic stress-related proteins in sporadic Creutzfeldt–Jakob disease (CJD) patients. (A) Detailed information on sporadic CJD patients and age- and sex-matched controls investigated in this study. (B) The expression patterns of endogenous proteolytic stress-related proteins were compared in the frontal cortex between sporadic CJD patients and matched healthy controls by western blotting. (C) Quantification of the expression levels of endogenous proteolytic stress-related proteins was normalized to the *β*-actin level. CTL: Age-matched healthy controls, *n* = 3; CJD: CJD patients, *n* = 3. *⁣*^*∗*^*p*  < 0.05; *⁣*^*∗∗*^*p* < 0.01; *⁣*^*∗∗∗*^*p*  < 0.001; N.S., not significant.

We also investigated the expression pattern of proteolytic stress-related proteins in the frontal cortex of sporadic CJD patients and matched controls using western blotting (Figure 2). Detailed information on sporadic CJD patients and matched controls is described in Figure 2A. In brief, the expression level of PrP was similar between sporadic CJD patients and matched controls. However, PrP^Sc^ was detected in only sporadic CJD patients. Similar to the western blotting results in ME7 scrapie-infected mice, the expression levels of CD10, cathepsin B, cathepsin D, and MMP9-40 kDa were upregulated in sporadic CJD patients. However, MMP9-90 kDa was downregulated in sporadic CJD patients (Figure 2B,C).

### 3.3. In Vitro Evaluation of the PrP^Sc^ Degradation Capacity of Nattokinase and Lumbrokinase

**Figure 3:**
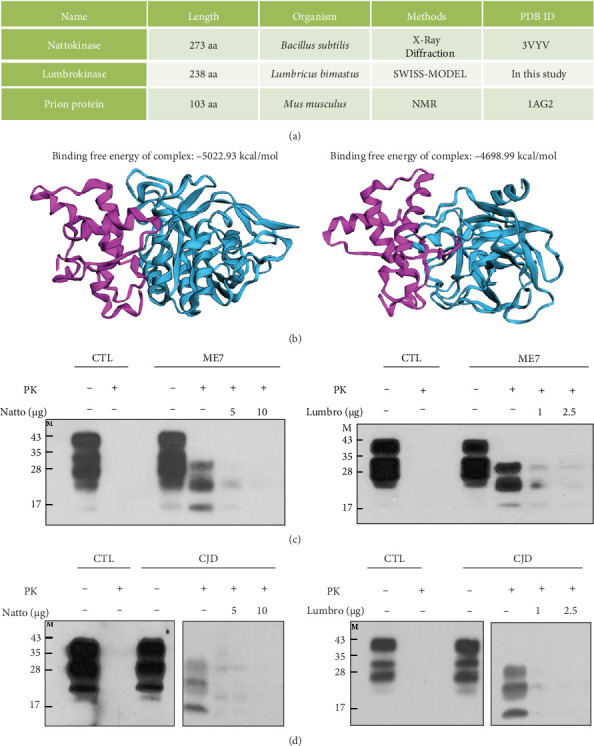
In vitro evaluation of the capacity of proteinase K-resistant prion protein (PrP^Sc^) degradation by nattokinase and lumbrokinase. (A) Detailed information on nattokinase, lumbrokinase, and murine prion protein (PrP). (B) The 3D structure and protein–protein interaction complex of murine PrP with nattokinase and lumbrokinase. The left panel indicates the interaction of murine PrP with nattokinase. The right panel indicates the interaction of murine PrP with lumbrokinase. The purple structure indicates murine PrP. The cyan structures indicate nattokinase (left panel) and lumbrokinase (right panel). (C) In vitro evaluation of the capacity of PrP^Sc^ degradation by nattokinase and lumbrokinase in ME7 scrapie PrP^Sc^. The left panel indicates western blotting results for the capacity of PrP^Sc^ degradation by nattokinase. The right panel indicates western blotting results for the capacity of PrP^Sc^ degradation by lumbrokinase. CTL: Brain homogenate derived from phosphate-buffered saline (PBS)-inoculated mice; ME7: brain homogenate derived from ME7 scrapie-inoculated mice. (D) In vitro evaluation of the capacity of PrP^Sc^ degradation by nattokinase and lumbrokinase in sporadic CJD PrP^Sc^. The left panel indicates western blotting results for the capacity of PrP^Sc^ degradation by nattokinase. The right panel indicates western blotting results for the capacity of PrP^Sc^ degradation by lumbrokinase. CTL: Brain homogenate derived from age-matched healthy controls; CJD: brain homogenate derived from sporadic CJD patients. PK: proteinase K; −: proteinase K-untreated lane; +: proteinase K-treated lane.

To evaluate the stable binding of nattokinase and lumbrokinase with PrP, we performed an in silico evaluation of the binding complex of murine PrP with nattokinase and lumbrokinase and calculated the binding free energy. Detailed information on nattokinase, lumbrokinase, and murine PrP is presented in Figure 3A, and the 3D structure of the binding complex is shown in Figure 3B. The binding free energy of murine PrP with nattokinase (−5022.93 kcal/mol) was lower than that of murine PrP with lumbrokinase (−4698.99 kcal/mol). The binding free energies of the two enzymes indicate a strong interaction, with the difference suggesting that nattokinase may have a slightly stronger affinity for murine PrP compared to lumbrokinase.

To evaluate the degradation capacity of scrapie PrP^Sc^ by nattokinase and lumbrokinase, we treated brain homogenates derived from ME7 scrapie-inoculated mice with proteinase K. The proteinase K-treated samples were treated with nattokinase and lumbrokinase to evaluate the PrP^Sc^ degradation ability of these enzymes. Notably, PrP^Sc^ was significantly decreased in nattokinase- and lumbrokinase-treated brain homogenates derived from ME7 scrapie-inoculated mice compared to PBS-treated brain homogenates derived from ME7 scrapie-inoculated mice (Figure 3C, Supporting Information 1: Figure S1).

We also evaluated the degradation capacity of sporadic CJD PrP^Sc^ by nattokinase and lumbrokinase. The proteinase K-treated samples were treated with nattokinase and lumbrokinase to evaluate the PrP^Sc^ degradation ability of these enzymes. Similar to the results of scrapie PrP^Sc^, PrP^Sc^ was significantly decreased in nattokinase- and lumbrokinase-treated brain homogenates derived from sporadic CJD compared to PBS-treated brain homogenate derived from sporadic CJD (Figure 3D).

### 3.4. In Vivo Evaluation of the Protective Effects of Nattokinase and Lumbrokinase in ME7 Scrapie-Infected Mice

**Figure 4:**
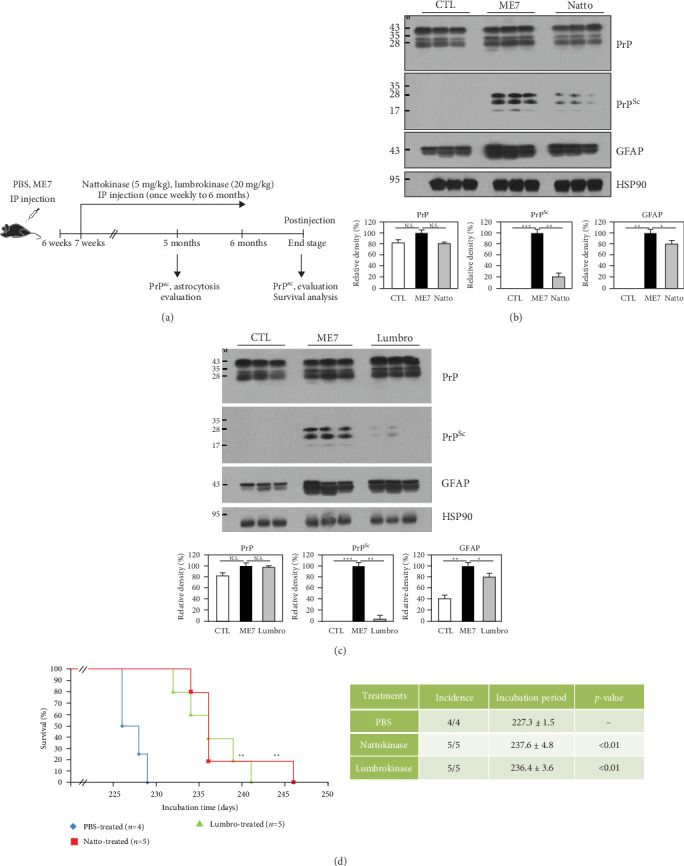
Evaluation of the protective effects of nattokinase and lumbrokinase in ME7 scrapie-infected mice. (A) Experimental design for the evaluation of the protective effects of nattokinase and lumbrokinase against prion disease in ME7 scrapie-infected mice. (B) Evaluation of the protective effects of nattokinase in ME7 scrapie-infected mice at 5 months postinjection. The upper panel indicates western blotting results. The lower panel indicates the quantification of the expression levels of proteinase K-resistant prion protein (PrP^Sc^) and astrocytosis-related protein normalized to the HSP90 level. Western blotting results were obtained from mouse brain tissue. CTL: Phosphate-buffered saline (PBS)-inoculated mice, *n* = 3; ME7: ME7 scrapie-inoculated mice, *n* = 3; Natto: nattokinase-treated ME7 scrapie-inoculated mice, *n* = 3. (C) Evaluation of the protective effects of lumbrokinase in ME7 scrapie-infected mice at 5 months postinjection. The upper panel indicates western blotting results. The lower panel indicates the quantification of the expression levels of PrP^Sc^ and astrocytosis-related protein normalized to the HSP90 level. CTL: PBS-inoculated mice, *n* = 3; ME7: ME7 scrapie-inoculated mice, *n* = 3; lumbro: lumbrokinase-treated ME7 scrapie-inoculated mice, *n* = 3. (D) Survival analysis in PBS-treated ME7 scrapie-inoculated mice (*n* = 4), nattokinase-treated ME7 scrapie-inoculated mice (*n* = 5), and lumbrokinase-treated ME7 scrapie-inoculated mice (*n* = 5). The left panel indicates the survival rate versus days after intraperitoneal inoculation. The right panel shows the summary of Kaplan–Meier survival analysis results. Statistical significance was calculated using the log-rank test. *⁣*^*∗*^*p* < 0.05; *⁣*^*∗∗*^*p* < 0.01; *⁣*^*∗∗∗*^*p* < 0.001.

To assess the protective effects of nattokinase and lumbrokinase against prion disease, we injected the ME7 scrapie strain into 6-week-old mice, treated them with PBS, nattokinase, and lumbrokinase weekly, and sacrificed them at 5 months postinjection (Figure 4A). We then evaluated PrP^Sc^ accumulation and astrogliosis in mouse brain tissue. Notably, PrP^Sc^ was significantly decreased in nattokinase- and lumbrokinase-treated ME7 scrapie-infected mice compared to PBS-treated ME7 scrapie-infected mice at 5 months postinjection. In addition, the astrocyte marker GFAP was also significantly reduced in nattokinase- and lumbrokinase-treated ME7 scrapie-infected mice (Figure 4B,C).

Furthermore, we carried out a survival analysis to evaluate the protective effects of nattokinase and lumbrokinase against prion disease (Figure 4D). Notably, the nattokinase (237.6 ± 4.8 days) and lumbrokinase (236.4 ± 3.6 days)-treated ME7 scrapie-infected mice showed significantly prolonged survival times compared to PBS-treated ME7 scrapie-infected mice (227.3 ± 1.5; Figure 4D). We also investigated PrP^Sc^ in the brains of nattokinase- and lumbrokinase-treated ME7 scrapie-infected mice at the end stage of prion disease. PrP^Sc^ accumulation was observed in both nattokinase- and lumbrokinase-treated ME7 scrapie-infected mice (Supporting Information 1: Figure S2).

## 4. Discussion

In the present study, we identified an abnormal expression pattern of proteolytic stress-related proteins in ME7 scrapie-infected mice and sporadic CJD patients (Figures 1 and 2). As expected, the elevated accumulation of endogenous proteases was observed in ME7 scrapie-infected mice and sporadic CJD patients. Previous studies have reported a clear association between proteolytic stress and the accumulation of endogenous proteases [18–20]. Thus, the brains of ME7 scrapie-infected mice and sporadic CJD patients are expected to have high proteolytic stress. However, due to the limited number of proteolytic stress markers used in this study, it is necessary to conduct high-throughput studies using mass spectrometry in the future to obtain more comprehensive results. In addition, further investigation of the relationship between proteolytic stress and the accumulation of endogenous proteases using knockout mice is highly desirable in the future.

Recent studies have reported that endogenous proteases, including CD10, MMP9, cathepsin D, and cathepsin B, showed fibrinolytic capacity to amyloid-beta fibrils of Alzheimer's disease [15, 16]. In addition, neurosin, the MMP family, cathepsin D and plasmin also showed fibrinolytic activity against alpha-synuclein in Parkinson's disease [17]. Thus, several studies have been performed to identify the therapeutic potential of endogenous proteases for Alzheimer's and Parkinson's diseases. However, since PrP^Sc^ has proteinase-resistant characteristics, these endogenous proteases are not appropriate for prion diseases. Thus, we introduced potent proteolytic enzymes, including nattokinase and lumbrokinase, to fundamentally reduce proteolytic stress by degrading PrP^Sc^. The interaction between PrP and nattokinase or lumbrokinase was predicted through in silico analysis (Figure 3B). However, to validate this interaction accurately, direct binding studies using in vitro techniques such as pull-down assays, isothermal titration calorimetry (ITC), and surface plasmon resonance (SPR) will be necessary in future experiments. Notably, scrapie and sporadic CJD PrP^Sc^ aggregates were significantly degraded by nattokinase and lumbrokinase (Figure 3C,D). In addition, we observed protective effects of nattokinase and lumbrokinase against prion disease in ME7 scrapie-infected mice (Figure 4). These results indicated that nattokinase and lumbrokinase have therapeutic potential for prion diseases. Although nattokinase and lumbrokinase did not perfectly protect against prion diseases, combination therapy with previously developed prion drugs seems to be highly desirable for the development of drugs for prion diseases. In addition, further studies on human native proteases capable of degrading PrP^Sc^ as potential gene therapy targets for prion diseases are highly desirable in the future. Due to the long-term nature of prion disease experiments, dose-dependent and combination studies for nattokinase and lumbrokinase were not conducted in this research. Future studies with varied concentrations will be necessary to fully assess their therapeutic potential.

In the present study, we focused on the degradation capacity of PrP^Sc^ by nattokinase and lumbrokinase. However, nattokinase and lumbrokinase are also associated with the plasminogen-related signaling pathway. Nattokinase and lumbrokinase promote tissue plasminogen activator (t-PA), and t-PA converts plasminogen to plasmin [28, 29]. Previous studies have reported that plasminogen plays a key role in the acceleration of prion disease progression [30–33]. Thus, further investigation on the mechanism of action (MOA) of nattokinase and lumbrokinase regarding plasminogen is needed.

## 5. Conclusions

In conclusion, we identified abnormal accumulation of proteolytic stress-related proteins, including CD10, cathepsin B, cathepsin D, and MMP9, in the brains of ME7 scrapie-infected mice and sporadic CJD patients. In addition, we found stable binding of nattokinase and lumbrokinase to PrP. Furthermore, we identified the potent degradation capacity of nattokinase and lumbrokinase for PrP^Sc^ derived from ME7 scrapie-infected mice and sporadic CJD patients. Finally, we identified the protective effects of nattokinase and lumbrokinase against prion disease in ME7 scrapie-infected mice. To the best of our knowledge, this is the first report on the identification of proteolytic stress-related novel potential biomarkers and the therapeutic potential of nattokinase and lumbrokinase for prion diseases.

## Data Availability

All data are available from the corresponding authors upon reasonable request.

## Supporting Information

Supporting Information 1 Figure S1: Quantification of PrP^Sc^ levels from the western blot results shown in Figure 3C. PK: proteinase K; -: proteinase K-untreated lane; +: proteinase K-treated lane; Natto: nattokinase; Lumbro: lumbrokinase. Figure S2: Western blotting detection of PrP^Sc^ in nattokinase- and lumbrokinase-treated ME7 scrapie-inoculated mice at the end stage. (A) Western blotting detection of PrP^Sc^ in nattokinase-treated ME7 scrapie-inoculated mice. (B) Western blotting detection of PrP^Sc^ in lumbrokinase-treated ME7 scrapie-inoculated mice. PK: proteinase K; -: proteinase K-untreated lane; +: proteinase K-treated lane.

Supporting Information 2 Table S1: Summary of the antibodies used.
