# Supplementary material for: Abnormal Expression of Proteolytic Stress-Related Proteins and Protective Effect of Fibrinolytic Enzymes in Prion Diseases
Source: Transbound Emerg Dis. 2025 Feb 26;2025:9527934. doi: 10.1155/tbed/9527934 (PMC12017092; doi:10.1155/tbed/9527934)

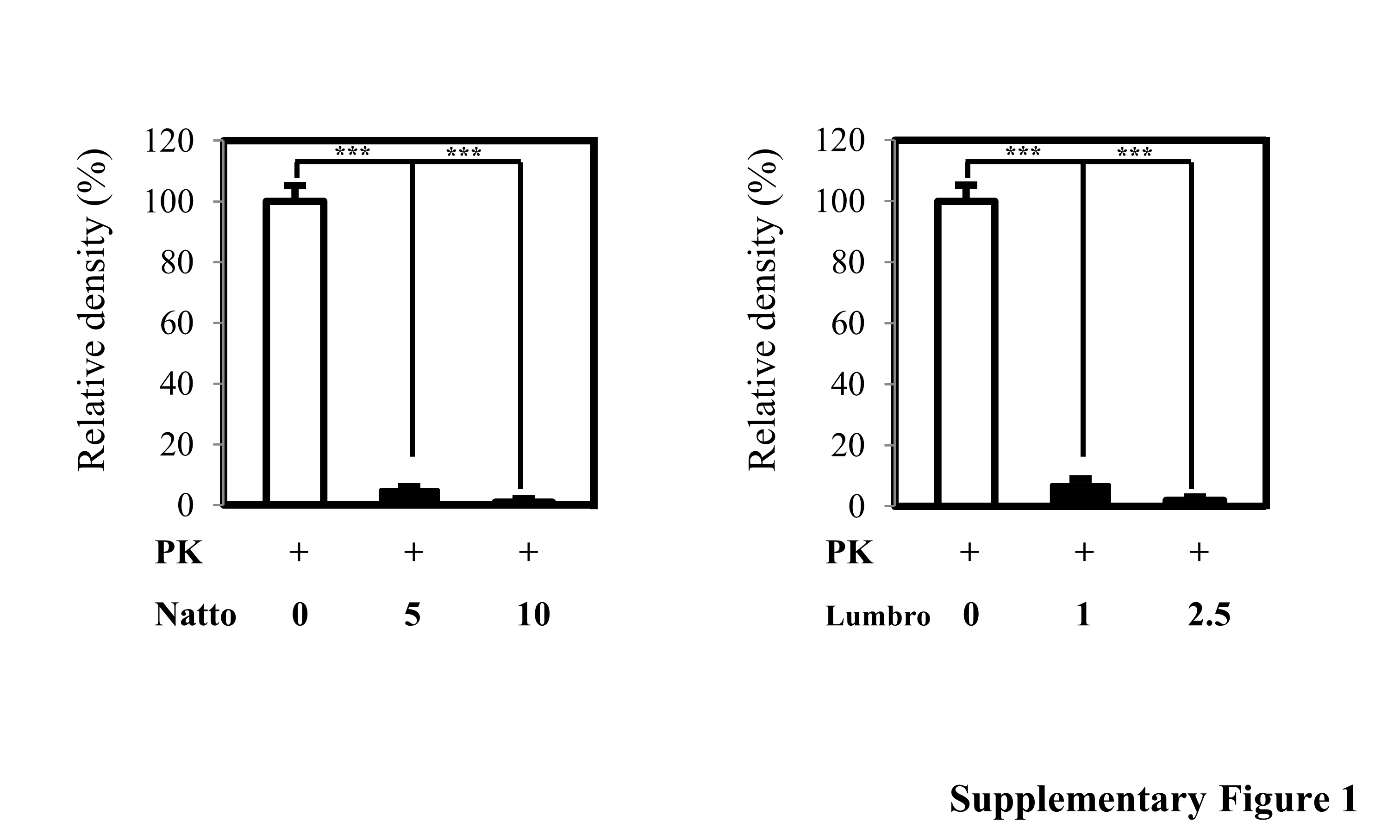


**Supplementary Figure 1** Quantification of PrP^Sc^ levels from the western blot results shown in Figure 3C. PK: proteinase K; -: Proteinase K-untreated lane; +: Proteinase K-treated lane; Natto: nattokinase; Lumbro: lumbrokinase.

**Supplementary Figure 2** Western blotting detection of PrP^Sc^ in nattokinase- and lumbrokinase-treated ME7 scrapie-inoculated mice of at the end stage. (A) Western blotting detection of PrP^Sc^ in the brain tissue of nattokinase-treated ME7 scrapie-inoculated mice. (B) Western blotting detection of PrP^Sc^ in the brain tissue of lumbrokinase-treated ME7 scrapie-inoculated mice. PK: proteinase K; -: Proteinase K-untreated lane; +: Proteinase K-treated lane.


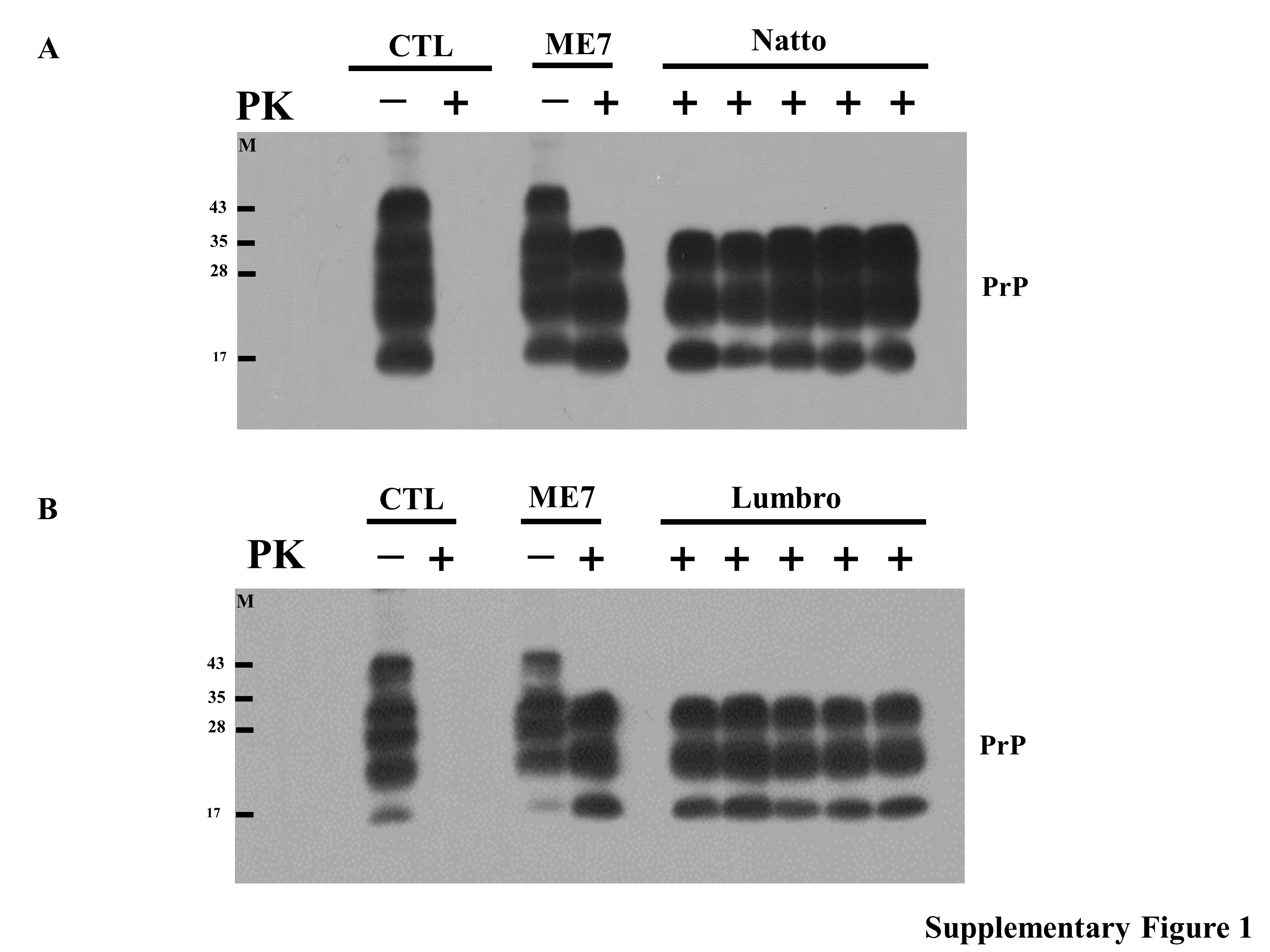

Supplement: Supporting Information 1 — Figure S1: Quantification of PrPSc levels from the western blot results shown in Figure 3C. PK: proteinase K; -: proteinase K-untreated lane; +: proteinase K-treated lane; Natto: nattokinase; Lumbro: lumbrokinase. Figure S2: Western blotting detection of PrPSc in nattokinase- and lumbrokinase-treated ME7 scrapie-inoculated mice at the end stage. (A) Western blotting detection of PrPSc in nattokinase-treated ME7 scrapie-inoculated mice. (B) Western blotting detection of PrPSc in lumbrokinase-treated ME7 scrapie-inoculated mice. PK: proteinase K; -: proteinase K-untreated lane; +: proteinase K-treated lane. [file 9527934.f1.docx]
