# Supplementary material for: Abnormal Expression of Proteolytic Stress-Related Proteins and Protective Effect of Fibrinolytic Enzymes in Prion Diseases
Source: Transbound Emerg Dis. 2025 Feb 26;2025:9527934. doi: 10.1155/tbed/9527934 (PMC12017092; doi:10.1155/tbed/9527934)
Supplement: Supporting Information 2 — Table S1: Summary of the antibodies used. [file 9527934.f2.docx]

**Supplementary Table 1** Summary of the antibodies used.

| Abbreviation | Name | Company | Reference number | Target species | Application (dilution) |
| --- | --- | --- | --- | --- | --- |
| SAF84 | Prion protein monoclonal antibody | Bertin | A03208 | Mouse | WB (1:200) |
| 3F4 | Prion protein monoclonal antibody | Enzo | ENZ-ABS119 | Human | WB (1:200) |
| GFAP | GFAP (2E1) | Santa cruz | Sc-33673 | Mouse | WB (1:100), IHC (1:50) |
| CD10 | Anti-CD10 antibody | Abcam | Ab256494 | Mouse, Human | WB (1:1000), IHC (1:100) |
| Cathepsin B | Anti-Cathepsin B antibody | Abcam | Ab58802 | Human | WB (1:250) |
| Cathepsin B | Anti-Cathepsin B antibody | Abcam | Ab214428 | Mouse | WB (1:1000), IHC (1:200) |
| Cathepsin D | Cathepsin D (D-7) | Santa cruz | Sc-377299 | Mouse, Human | WB (1:100), IHC (1:100) |
| MMP9 | Anti-MMP9 antibody | Abcam | Ab38898 | Mouse, Human | WB (1:1000), IHC (1:1000) |
| HSP90 | Purified Mouse Anti-Hsp90 | BD transduction laboratory | 610418 | Mouse | WB (1:200) |
| β-Actin | β-Actin (C4) | Santa cruz | Sc-47778 | Human | WB (1:200) |

WB: western blot; IHC: Immunohistochemistry
